# Novel pH-responsive and self-assembled nanoparticles based on *Bletilla striata* polysaccharide: preparation and characterization

**DOI:** 10.1039/c8ra07202g

**Published:** 2018-12-03

**Authors:** Junxiao Zhu, Xiaoxi Guo, Tingting Guo, Ye Yang, Xiuming Cui, Jun Pan, Yuan Qu, Chengxiao Wang

**Affiliations:** Faculty of Life Science and Technology, Kunming University of Science and Technology Kunming 650500 China wcx1192002@126.com; Key Laboratory of Sustainable Utilization of Panax Notoginseng Resources of Yunnan Province Kunming 650500 China; University Based Provincial Key Laboratory of Screening and Utilization of Targeted Drugs Kunming 650500 China; Institute of Food Science and Technology, Yunnan Provincial Academy of Agricultural Sciences Kunming 650205 China

## Abstract

In this investigation, innovative pH-sensitive and amphiphilic nanoparticles (NPs) were synthesized by grafting histidine (His, pH sensitive molecule) and stearic acid (SA, hydrophobic segment) onto the polysaccharides of *Bletilla striata* (BSP). The His-SA-BSP was able to self-assemble into NPs with pH sensitivity. The acidic conditions could trigger the imidazole ionization and reverse the surface charge, while the electrostatic repulsion wrecked the structure and drove the NPs to a swollen state, as revealed by dynamic light scattering (DLS), transmission electron microscopy (TEM), and critical micelle concentration (CMC) analyses. By increasing the degree of substitution (DS) of His, the NPs showed improved pH sensitivity. The NPs could accelerate Doxorubicin (Dox) release to a remarkably greater extent (3-fold) at pH 5 than at pH 7.4. The CCK-8 assay demonstrated a good biocompatibility of the NPs towards different cell lines and a specific inhibition effect of Dox-loaded NPs against tumor cells. Furthermore, the NPs showed the improved cellular uptake of Dox towards MCF-7 by fluorescence microscopy and flow cytometry. Therefore, the new His-SA-BSP showed potential applications in drug nanocarrier systems.

## Abstract 2

In this investigation, innovative pH-sensitive and amphiphilic nanoparticles (NPs) were synthesized by grafting histidine (His, pH sensitive molecule) and stearic acid (SA, hydrophobic segment) onto the polysaccharides of *Bletilla striata* (BSP).

## Graphical abstract

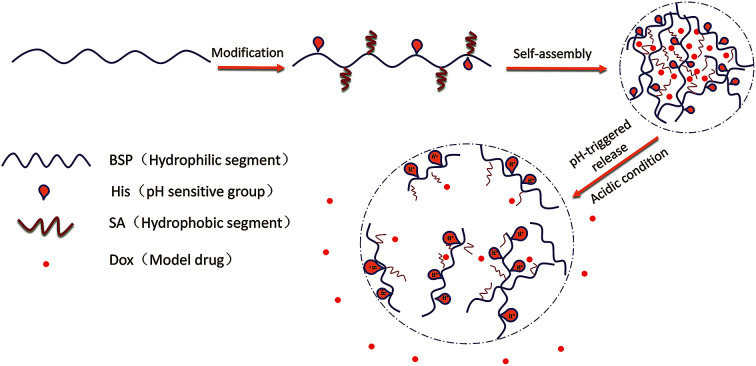

## Introductions

Polysaccharides have attracted increasing attention as smart nanoparticles (NPs)^1,2^ in drug delivery systems, particularly since they can be obtained in a well characterized and reproducible way from natural sources.^3^ Due to their biodegradable and hydrophilic nature, PS are usually grafted with small hydrophobic molecules or polymers to form the amphipathic skeleton of the NPs. Additionally, the varieties of the active groups (hydroxyl, amine, and carboxylic acid groups) existing along the PS chains can be modified with different substituents to provide various and multiple functions.^4,5^ If properly designed, smart self-assembly NPs can be prepared with the desired sizes and endogenous/extraneous stimuli-response properties for targeted drug delivery.^6,7^

*Bletilla striata* polysaccharides (BPS) are extracts from the tubers of *Bletilla striata*, which is a neutral water-soluble glucomannan known to exhibit the therapeutic effects of anti-fibrosis,^8^ anti-tyrosinase^9^ and immunoregulation.^10,11^ It is widely accepted that BPS predominantly consists of glucomannan, with a backbone of (1 → 4)-β-d-mannose and glucose at molar ratio of 3 : 1.^12^ This polysaccharide is a promising material for drug delivery systems. By physical blending or crosslinking, BSP could be introduced into the drug delivery carriers of microspheres,^13^ hydrogels^14^ and so on. The abundant hydroxyl groups in BPS allowed the modification of either the functional molecules^10,15^ or active ingredients.^16^ Among these, the amphiphilic grafted BSP is highly valued for the self-assembly properties. By linking cholesteryl succinate^17^ or fatty acid^18^ to BSP, amphiphilic NPs could be formed in aqueous solutions within the size range of 250 to 400 nm. The NPs possessed hepatic targeting capabilities^19^ and were designed as the nanocarriers for cancer therapy.^20,21^ Furthermore, the BSP derivatives can be used for cancer immunotherapy due to its affinity to the tumor associated macrophages.^16,22^

**Fig. 1:**
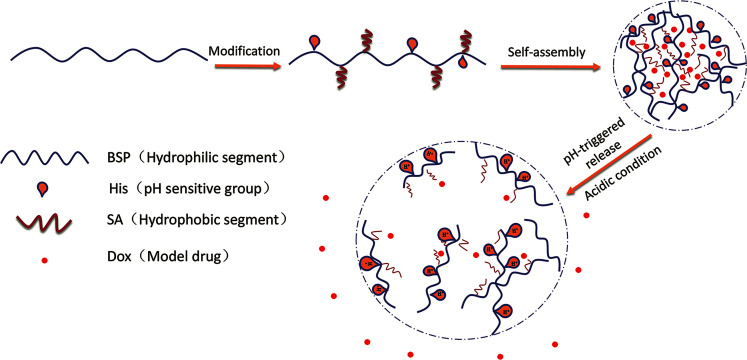
Schematic illustrations of self-assembly His-SA-BSP NPs and pH responsive Dox delivery.

In our previous work, BSP was prepared and used as a framework of hydrogel. The examined BSP-hydrogel had displayed skin permeation enhancements and hemostatic activities for transdermal drug delivery.^23^ In the current study, ingenious NPs with pH sensitive profiles were designed. The purified BSP were first modified by histidine (His-BSP), a commonly used function molecular for pH sensitivity.^2,24,25^ After that, stearic acid (SA) was introduced into the chain using a simple esterification method in order to provide the hydrophobic segment.^18^ The obtained His-SA-BSP were evaluated including self-assembly behaviors, pH sensitivities and pH-triggered release profiles of Doxorubicin (Dox) (Fig. 1). Additionally, cytotoxicity assays as well as the *in vitro* cellular uptake of Dox were performed. The results of this study can potentially provide valuable data and approaches for the molecular designs of novel NPs based on the natural polysaccharides.

## Materials and methods

### Materials

The dried tubers of *Bletillastriata* were provided by the Liangbao Co., Ltd. (Yunnan, China). The l-histidine (l-His, >99%); 1-ethyl-3-(3-dimethylaminopropyl) carbodiimide (EDC, >98.5%); *N*-hydroxysuccinimide (NHS, >98.5%); *N*,*N*′-dicyclohexylcarbodiimide (DCC, >99%); and dimethylaminopyridine (DMAP, >99%) were purchased from the Sigma Chemical Co. (USA). Doxorubicin was purchased from Shanghai HuaLan Chemical Technology Co., Ltd, China (USP, ≥98%, DB3456-100 mg). The other chemicals used in this study were of analytic grade and supplied by the Sinopharm Chemical Reagent Co., Ltd (Shanghai, China).

### Preparation of the BSP

The extraction and purification processes of BSP was referred to our previous work with some modifications.^23^ In brief, the tubers (200 g) were smashed (YC-06B micronizer, Jinben, China) and thermo-extracted twice in 4 L distilled water (70 °C, 3 hours). After filtration, the aqueous extracts were condensed to a 1/3 volume of the primary liquid by a rotary evaporator (N-1300S-W, EYELA, Japan) and then precipitated with 95% ethanol to a final concentration of 75%. The precipitates were collected by a centrifuge (H1850R, Hunan Xiangyi Co., Ltd. China) to obtain the crude BSP. The protein was removed by the Sevag reactions. After that, the BSP was applied to a DEAE–(Cl^−^)–cellulose-52 (4 × 40 cm) column and a Sephadex G-200 (1.6 × 80 cm) column for purification (0.1 mol L^−1^ NaCl, 20 mL h^−1^). After dialysis (MWCO 3500, Sigma) and lyophilization (Scientz-10ND, Ning bo Scientz Co., Ltd. China), the purified BSP was obtained.

### Synthesis of the His-SA-BSP

**Fig. 2:**
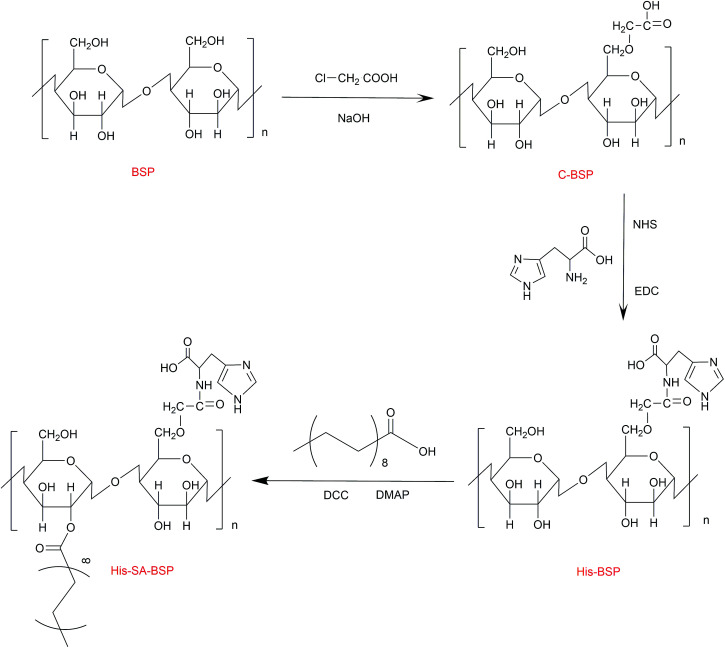
Synthetic route of His-SA-BSP.

The synthesis scheme of the His-SA-BSP was shown in Fig. 2, which included three steps as follows: first, the C-6 hydroxyl group of the monosaccharide units was activated by carboxymethylation reaction; second, the l-histidine was reacted with the carboxymethyl group; and the third step was the grafting of the fatty acid chain onto the His-BSP by esterification.

#### Carboxymethylation

The purified BSP products were dissolved in 10 mL water to a homogeneous colloidal solution, then NaOH (30 mL, 5 mol L^−1^) and chloroacetic acid (5 g) were separately added and reacted (60 °C, 3 hours). Then the mixture was adjusted to pH 7.0 and then dialyzed (1 day) to obtain the carboxymethylation BSP (C-BSP).

#### l-Histidine modification

The obtained C-BSP was dissolved in 15 mL water. Then, certain amounts of EDC and NHS were added as condensing and coupling agents, respectively. The reaction mixture was stirred (25 °C, 2 hours) to activate the carboxyl groups. Then, l-histidine was added and reacted (25 °C, 24 hours). After that, the mixture was dialyzed and then freeze dried to obtain the His-BSP.

#### Esterification

The His-BSP was dispersed in 20 mL of DMSO and stirred. SA (1.0 mmol) was added into 5 mL DMSO and activated by DCC (1.0 mmol) and DMAP (1.2 mmol) for 2 hours. The resulting mixture was added drop wise to the His-BSP solution and stirred (2 hours at 80 °C; and then 24 h at 25 °C). The reaction mixture was firstly dialyzed and then extracted with ethyl acetate. After lyophilization, the final His-SA-BSP product was obtained.

### Characterization

#### Fourier transform infrared spectral analysis

The sample was subjected to FTIR spectroscopy using a FTIR spectrometer (Nicoletis 10, Thermo Co., Ltd. USA) with scanning range of 4000 to 500 cm^−1^.

#### NMR spectroscopy

The samples were characterized by ^1^H-NMR and ^13^C-NMR spectra using D_2_O (BSP, C-BSP and His-BSP) or D-DMSO (His-SA-BSP) at concentrations of 30 to 50 mg mL^−1^. Then, the spectra were recorded on a Bruker ARX-600 instrument (600 MHz, Bruker Co., Ltd. Switzerland).

#### Molecular weight

HPGPC was used to determine the homogeneity and molecular weight of the BSP. In summary, a 2 mg mL^−1^ sample was loaded onto HPLC (waters 515 and 2414 refractive index detector) equipped with an ultra-hydrogel series column (7.8 mm × 300 mm). 20 mM CH_3_COONH_4_ solution was used as the eluent at a flow rate of 0.5 mL min^−1^. The column was calibrated using the Dextran standards of different molecular weights.

#### Degree of substitution (DS)

The degree of substitution (DS) of His was determined based upon the DS of carboxymethyl and the C/N ratio of His-BSP.^26^ Briefly, the DS of carboxymethyl was determined using a neutralization titration method,^27,28^ while the C/N ratio was directly recorded by the CHN elemental analyzer (Vario EL III, Elementar Co., Ltd. Germany).^29^

#### Micromorphology

The micromorphology of the BSP powder and the obtained products were observed using Scanning Electron Microscope (Quanta 450, FEI Co., Ltd. USA).

### Preparation of the NPs

#### Blank sample

The self-assembled NPs were prepared using an ultrasonic method as follows: first, 25 mg of the His-SA-BSP products were dispersed into 10 mL water and stirred (37 °C, 4 hours) until completely dissolved; then, the solution was treated using a ultrasonic processor (JY99-II, Scientz, Ningbo, China) at 200 W for 10 minutes. The resulting suspension was then filtered through a 0.45 μm syringe filter, and used for the immediate analysis.

#### Dox-loaded NPs

30 mg of triethylamine (TEA) was added into 10 mL DMSO solution of DOX. HCL (0.3 mg mL^−1^) to remove the hydrochloride. 6 mg His-SA-BSP was dispersed in 4 mL DMSO and then added to the above solution. The mixture was stirred (37 °C), followed by the dialysis against PBS (pH 7.4, 0.01 M) for 72 hour under dark conditions. Finally, the DOX-loaded NPs solution was lyophilized to obtain a pink powder. The products were dissolved in a certain volume of DMSO for the determinations of the entrapment efficiency (EE), and drug loading content (LC).^30,31^ The DOX concentration was analyzed by using UV-vis spectroscopy (480 nm).^32^
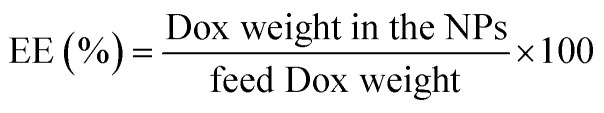

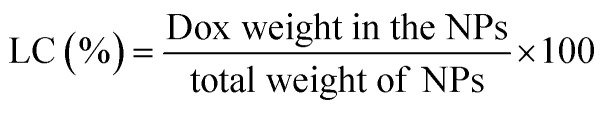


### Characterization

The morphologies of the NPs were observed using transmission electron microscopy (TEM). One droplet of NPs was placed on a carbon-coated copper grid, and the excess solution was removed with filter paper. Then, the morphology of the NPs was examined and photographed (JEM-2100, JEOL Ltd., Japan). In addition, the mean particle sizes, size distribution, as well as the zeta potential were determined by dynamic light scattering analysis (90 Plus, Brookhaven Instruments Co., USA).

### Critical micelle concentration (CMC)

The critical micelle concentrations (CMC) of the NPs were determined by fluorescence spectroscopy, using pyrene as a fluorescent probe. A certain concentration of pyrene–acetone solution was prepared and evaporated to remove the solvent. Then, 10 mL of the His-SA-BSP suspension at various concentrations were added and shaken overnight. The final concentration of pyrene was fixed at 1.0 × 10^−6^ mg mL^−1^. Then a fluorescence spectrophotometer (970CRT, Shanghai Precision Instrument Co., Ltd. China) was used to measure the steady-state fluorescence spectra with excitation wavelength of 332 nm and the emission spectra ranged from 320 to 450 nm. The ratio of the intensity at 373 nm (I373) to that at 390 nm (I390) was calculated and plotted against the common logarithm of the His-SA-BSP concentration. The CMC was determined at the turning point in the plot.

### XRD evaluation

The crystalline state of Dox, His-SA-BSP, physical mixture and drug-loaded NPs were measured by an X-ray powder diffraction instrument (XRD-6000, Shimadzu, Japan) at 40 mA and 40 kV. Standard runs were performed with a scanning rate of 0.02° min^−1^ over a 2*θ* range of 3 to 85°.

### Drug release profile

The Dox-NPs were weighed and dissolved in 5 mL of phosphate buffered saline (PBS) solution of various pH values (pH 7.4 and 5.0), and then transferred for dialysis. 25 mL of PBS (pH 7.4 or 5.0 respectively) was added into the outer solution to initiate the release (37 °C, 100 rpm). At predetermined time points, 3 mL of the release solution was collected and the same volume of fresh solution was added. The amount of released DOX was determined and the release curves were plotted.

### Cell culture

Three tumor cell lines, HepG2 (liver hepatocellular carcinoma cell line), MCF-7 (human breast adenocarcinoma cell line), HGC-27 (human gastric carcinoma cell line) and a normal cell lines, HL-7702 (normal liver cell line) were kindly provided by Yunnan Labreal Biotechnology Co., Ltd. The cells were cultured in DMEM or 1640 mediums at 37 °C in a humidity atmosphere with 5% CO_2_. The medium was supplemented with 10% FBS, 100 μg mL^−1^ streptomycin and 100 mL^−1^ penicillin.

### Cytotoxicity

CCK-8 (cell counting kit-8) assay was employed to assess the cytotoxicity of blank NPs against the four cells. Cells were seeded onto 96-well plates (2.5 × 10^3^ cells per well) and cultured for 24 hours. Then the cells were exposed to fresh medium containing series concentration of blank His-SA-BSP2 NPs and incubation for 48 hours. CCK-8 solution (10 μL) was then added into each well and further incubated (2 hours, 37 °C). A microplate reader (SM600, Shanghai UtraoMedical Instrument Co., Ltd) was employed to measure the absorbance (450 nm). The cell viability was calculated as follows:^20^
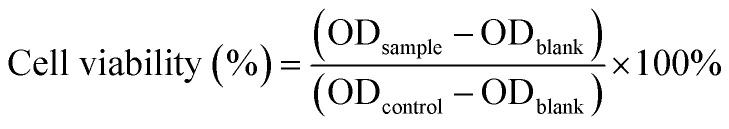
where OD_sample_ represented OD measured in cells under the treatment of NPs; OD_control_ represented the OD measured from the cells treated with incubation solution and OD_blank_ is the OD of incubation solution alone.

Moreover, the IC_50_ value of each group was then calculated using SPSS 19.0 (Chicago, IL, USA).

### Tumor cell inhibition

To evaluate the effect of Dox-loaded NPs on the tumor cell, the three tumor cells were co-cultured with the Dox-loaded NPs solution (His-SA-BSP2, containing 5 μg mL^−1^ or 10 μg mL^−1^ Dox). Dox solutions (5 μg mL^−1^ or 10 μg mL^−1^) were set as comparisons. The experiments were conducted using CCK-8 assay as detailed above.

### Cell uptake

#### Fluorescence microscopy

MCF-7 cells were selected as the model cell line and seeded in 24-well plates (1 × 10^4^ cells per well) and incubated (48 hours, 37 °C). Free Dox (10 μg mL^−1^) or Dox-loaded NPs solution (His-SA-BSP2, containing 10 μg mL^−1^ Dox) in serum-free medium was added and incubated for 0.5, 2 and 4 hour. Then, all reagents were removed and Hoechst 33 342 was used to visualize the nuclei (10 μg mL^−1^, 15 min).^25^ Afterwards, cells were examined by fluorescence microscopy (IX51, Olympus).

#### Flow cytometry

The cellular uptake of NPs was also analyzed quantitatively using flow cytometry. MCF-7 cells were seeded at a density of 1 × 10^4^ cells per well in 6-well plates and incubated for 24 h. After incubating with free DOX (10 μg mL^−1^) or DOX-loaded NPs (His-SA-BSP2, containing 10 μg mL^−1^ Dox) for 0.5, 2 and 4 hour, the cells were washed, harvested and subsequently resuspended in 0.5 mL PBS for flow cytometry analysis (PartecGmbH CyFlow Space). The mean fluorescence intensity of each test was recorded.

## Results and discussion

### Preparation and characterization of BSP

**Fig. 3:**
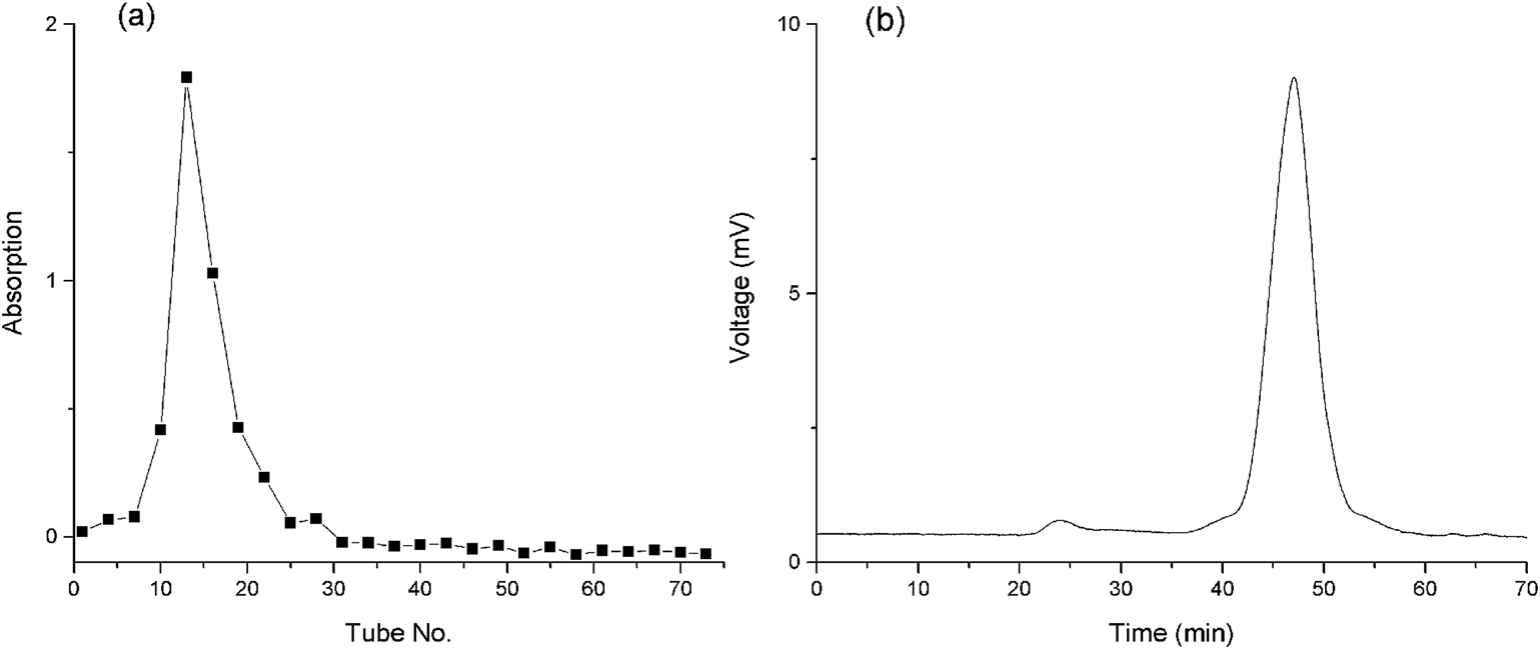
Purity of the BSP. (a) Outflow curve of BSP from g-100 column; (b) HPGPC spectrum of BSP.

Aqueous extractions reinforced by thermal treatments are commonly used as the extraction strategy of plant polysaccharides due to the economical nature and convenience of use.^33^ The extraction yield of crude BSP was 18.3%. After Sevag reaction, the BSP was further purified using DEAE and G-200 columns. A single and symmetrical peak was detected and collected (Fig. 3a) by the phenol-sulfuric acid method and HPGPC (Fig. 3b), which indicated a homogeneous material with molecular weight of 176 kDa. The yield of the final product was determined to be 33.8% based upon the crude BSP.

**Fig. 4:**
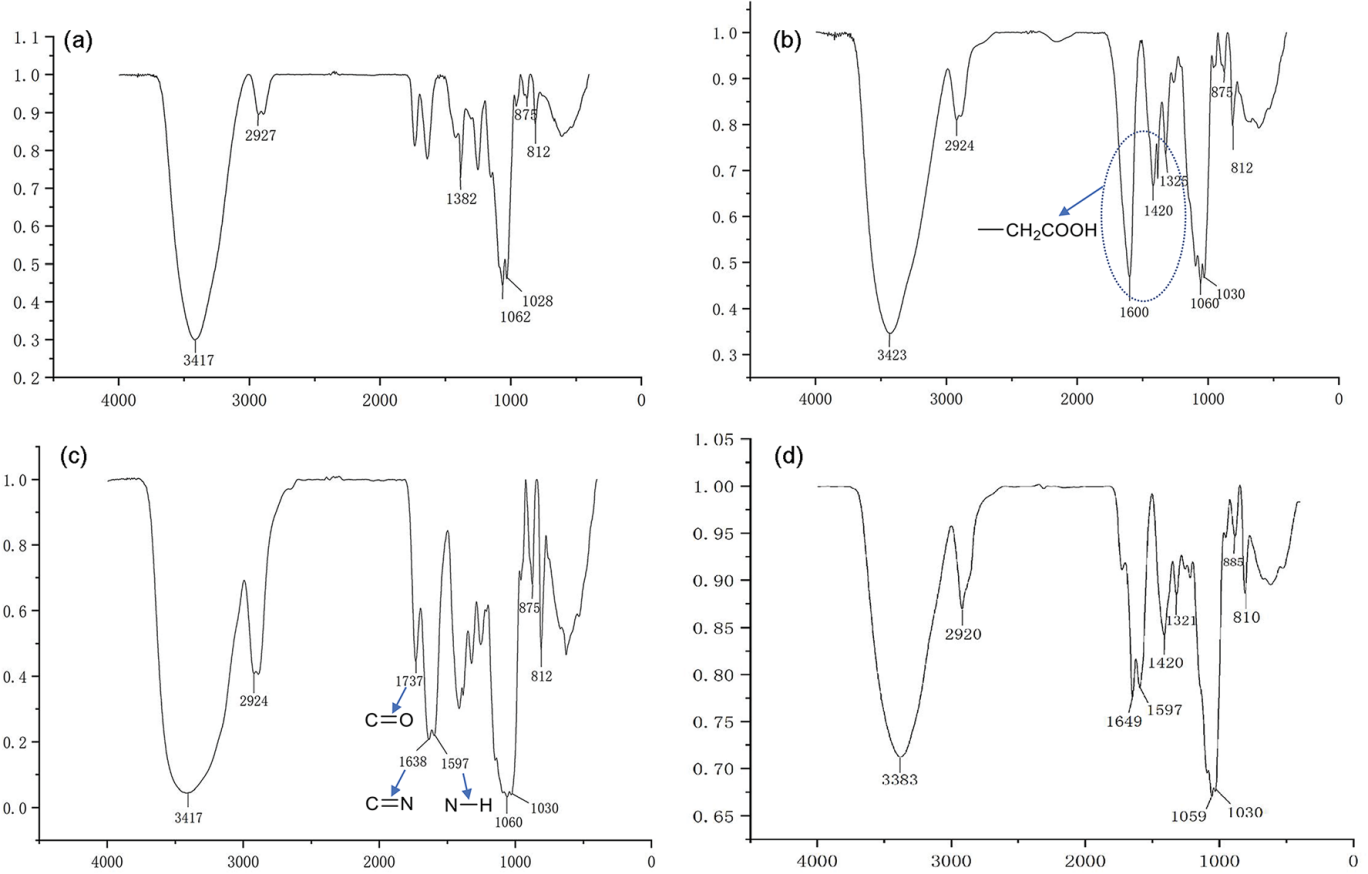
FT-IR characterization of BSP and its derivatives. (a) BSP; (b) C-BSP; (c) His-BSP; (d) His-SA-BSP.

**Fig. 5:**
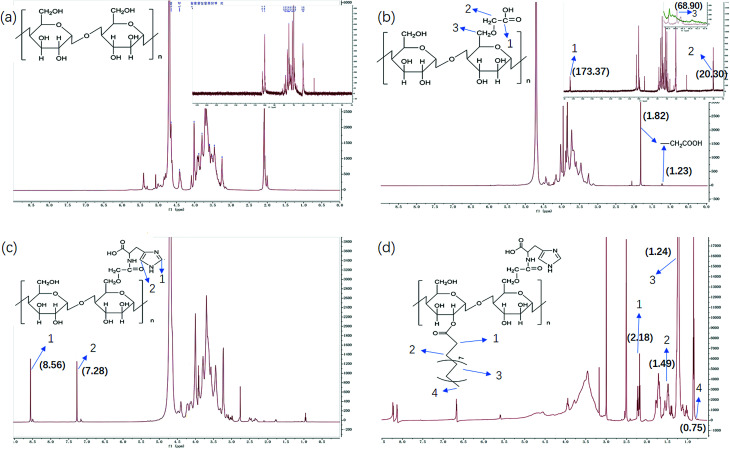
NMR characterization of BSP and its derivatives. (a) BSP; (b) C-BSP; (c) His-BSP; (d) His-SA-BSP.

We have demonstrated that the BSP was composed of mannose and glucose, with a relative mole ratio of 2.8 : 1.^23^ In the current work, the structural information of BSP was revealed by the FTIR (Fig. 4a) and NMR spectrums (Fig. 5a). The characteristic absorptions at 812 cm^−1^ and 875 cm^−1^ confirmed the existence of mannose (Kong *et al.* 2015). Additionally, the presence of pyranose was determined at 1028 cm^−1^. Further structural features were determined using ^13^C-NMR and ^1^H-NMR spectroscopy (Fig. 5a). Assignments of the chemical shifts were compared with the published data,^8,17^ and listed in Table 1.

**Table 1:** ^13^C-NMR and ^1^H-NMR spectral assignment of BSP

Sugar residues		Chemical shifts *δ* (ppm)					
		1	2	3	4	5	6
→4)-β-d-Man-(1→	^13^C	100.10	69.93	71.40	76.49	74.99	60.47
	^1^H	4.64	4.02	3.67	3.71	3.45	3.80
→4)-β-d-Glc-(1→	^13^C	102.47	72.18	75.15	78.44	74.63	60.08
	^1^H	4.41	3.24	3.60	3.51	3.59	3.88

### Synthesis and characterization of His-SA-BSP

#### C-BSP

The C-BSP was achieved using a classical approach as described in the previously work.^27,28,34^ As detailed in Fig. 5b, it can be certified in ^1^H-NMR spectrum as new signals at *δ* (ppm) 1.82 and 1.23.^27^ In the ^13^C-NMR spectrum, new signals at *δ* 173.37 ppm (C=O) and 20.30 ppm (–CH_2_–) evidenced the carboxymethylation reaction. Furthermore, the peaks of C6 shift from 60 to 68.90 ppm, while no shifts were observed for signals of C2–C5. It suggested that C6 was the primary carbon for the carboxymethylation reaction.^27,34^ In addition, the FT-IR peak (Fig. 4b) at 1600, 1420 and 1325 evidenced the existing of –CH_2_COOH.^27^

#### His-BSP

In the ^1^H-NMR spectrum (Fig. 5c) of His-BSP, the new peaks at *δ* (ppm) 7.28 and 8.56 were attributed to the –CH of the imidazole group.^2^ In FT-IR spectrum, the stretching vibration of C=N and N–H deformation vibration in His appeared at 1638 and 1597 cm^−1^,^35^ respectively. All the results suggested the successful synthesis of His-BSP.

His and its polymer are often used as the pH sensitive moieties in NPs.^2,24,36^ The His was pre-protected by *t*-butyloxycarbonyl and 2,4-dinitrophenol, and then subjected to esterification with the hydroxyl of the monose. Although the reaction was effective, the site of His could not be clearly assigned. Therefore, we provided a new strategy for His modification in the current study. The BSP was subjected to carboxymethylation in advance at in the C-6 site. Then, His can be introduced to the specific locations by the reaction between the amino and the carboxyl groups. By control the His/C-BPS ratio, different DS can be achieved.

#### His-SA-BSP

The SA modification was successfully achieved under a simple esterification.^18,19^ In the ^1^H-NMR spectrum (Fig. 5d), new peaks at *δ* (ppm) 2.18 and 1.49 were attributed to the methylene groups beside the carbonyl group of SA. The broadening peaks at *δ* (ppm) 1.24 suggested the other methylene groups of SA. The methyl in the end showed *δ* (ppm) of 0.75. C-2 and C-6 were the mainly reactive sites of glucosamine units. Since the carboxymethyl occupied the site of C-6, the reaction with SA was supposed to be taken place at the C-2 sites.^18^

#### Morphology

**Fig. 6:**
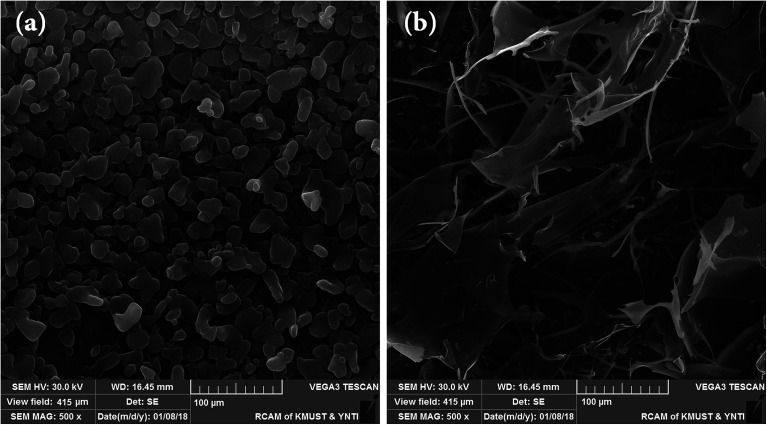
Scanning electron micrographs of (a) BSP and (b) His-SA-BSP at magnifications of 500×.

The morphologies of PS are directly related to the processing methods^27,37^ and their own characteristics.^28^ The BSP could be completely dissolved in the aqueous phase and presented a homogeneous nature and smooth appearance after lyophilization (Fig. 6a). However, the modified products had been subjected to phase separation in the aqueous solution due to their amphiphilic properties. Therefore, it presented irregular sheet with fibrotic structure after lyophilization (Fig. 6b).^27^

### Preparation and characterization of NPs

The His-SA-BSP underwent the entropy-driven self-assembly processes in aqueous solution, which was comprised of a double phase separation: the separation of the SA from the water, and the intra-molecular separation of the BSP from the hydrophobes. Geometrically, if a phase separation features a volumetric balance dominated by the hydrophile, high-curvature, water-dispersed objects, *i.e.* NPs will be formed.^38^ In the current work, His-SA-BSP with different His-DS was prepared and used (Table 2), namely His-SA-BSP1 (His-DS of 17.2%) and His-SA-BSP2 (His-DS of 29.7%).

**Table 2:** Characterization of the His-SA-BSP (*n* = 3, mean ± S.D)

	His-SA-BSP1					His-SA-BSP2				
	Size (nm)	PDI	Zeta potential (mV)	CMC (μg mL^−1^)	pH_d_	Size (nm)	PDI	Zeta potential (mV)	CMC (μg mL^−1^)	pH_d_
pH 7.4	194.31 ± 5.21	0.189 ± 0.016	−11.53 ± 1.17	70.79	4.9	214.99 ± 4.32	0.170 ± 0.014	−12.30 ± 1.08	36.98	5.2
pH 5.0	364.03 ± 7.14	0.195 ± 0.022	7.09 ± 0.84	27.54		427.59 ± 6.88	0.205 ± 0.019	12.36 ± 0.67	17.17	

#### DLS evaluation

**Fig. 7:**
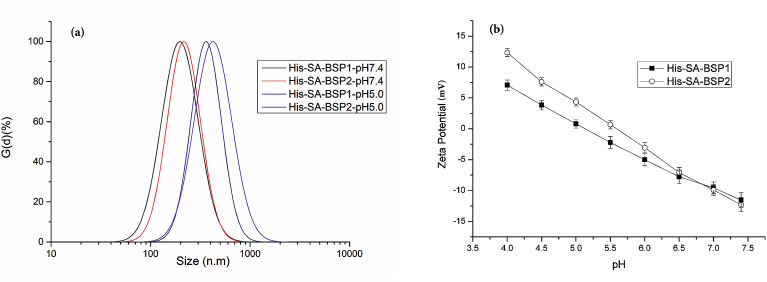
Dynamic light scattering analysis of His-SA-BSP NPs. (a) Size distribution; (b) zeta potential (*n* = 3).

DLS evaluations of the NPs were listed in Fig. 6a. Size is an essential parameter for preventing glomerular filtration and extravasation from the normal blood vessels.^35^ The particle size and PDI of His-SA-BSP1 were 194 ± 5 nm and 0.189 at pH of 7.4 respectively (Table 2), which indicated a narrow size distribution. When the pH reduced to 5.0, the sizes of the His-SA-BSP1 increased to approximately 364 ± 7 nm (Fig. 7a). By increasing the His-DS, the NPs showed improved pH-sensitivity. The sizes of the His-SA-BSP2 increased to 427 ± 6 nm at a pH 5.0, with the PDI of 0.205.

Zeta potential is crucial for the stability of the NPs. At a pH level of 7.4, the zeta potential of His-SA-BSP1 was approximately – 11 mV (Table 2 & Fig. 7b). When the pH decreased to 5.0, the value was reversed to 7 mV, implying that the NPs had the ability to change its zeta-potential in response to the environmental pH levels. For His-SA-BSP2, when the pH decreased from 7.4 to 5.0, the zeta potential reversed from −12 mV to 12 mV (Fig. 7b).

The pH-triggered changes in sizes and zeta potential were consistent with reported work.^2,39–41^ Guan *et al.* prepared pH-sensitive BSP micelles by stearic acid modification.^20^ The pH-sensitivity of the said micelles was attributed to the abundant negative charges carried by SA-BSP, along with the isoelectric point of the BSP. However, in our work, the pH-sensitive properties were mainly related to the specific His feature.^40^ His consists of an imidazole group, a carboxyl group, and an amino group with a pKa of 6.05.^40^ The electron lone pair on the unsaturated nitrogen of the imidazole ring made the His amphoteric by protonation–deprotonation.^2^ Therefore, the acidic conditions can trigger the imidazole ionization and drive the NPs into a swollen state. Once the ionized unimers repelled each other by the strong electrostatic repulsion, the NPs segments aggregated to the larger particles and became increasingly unstable.

#### Morphologies

**Fig. 8:**
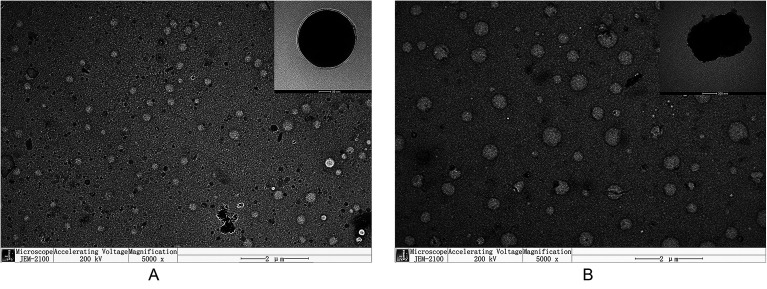
The morphology of His-SA-BSP2 measured by transmission electron microscope at different pH value. (A) pH 7.4 (B) pH 5.0.

The morphologies of the His-SA-BSP2 were observed by TEM (Fig. 8). The NPs were observed to be spherical and regular in shape at pH 7.4. However, at pH 5.0, the NPs had become swollen and damaged, which was consistent with the DLS results.

#### CMC

**Fig. 9:**
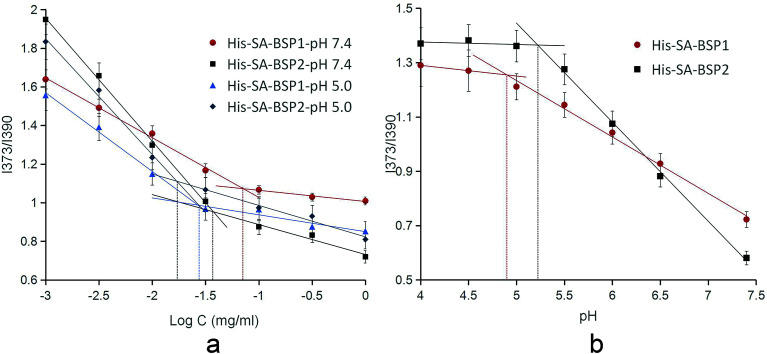
Critical micelle concentration (a) and acidic dissociation pH of the NPs (b) (*n* = 3).

The CMC of the NPs were calculated as detailed (Table 2 & Fig. 9a). The CMC of His-SA-BSP1 were 71 μg mL^−1^ and 28 μg mL^−1^ at pH 7.4 and 5.0, respectively. Due to the enhanced hydrophobic/hydrophilic balance resulted from the increased His segment,^25^ the CMC of His-SA-BSP2 decreased to 37 μg mL^−1^ and 17 μg mL^−1^ at pH 7.4 and 5.0, respectively.

The CMC is the main parameter to determine the thermodynamic stability of the micelles drug delivery systems.^42^ The CMC of the NPs were significantly lower than other surfactants,^43^ suggesting an improved structural integrity of NPs at low copolymer concentrations (even under extreme dilution) and a better stability.

The pH of disruption was referred to as the acidic dissociation pH (pH_d_) of the NPs.^35^ The pH_d_ were 4.9 for His-SA-BSP1 and 5.2 for His-SA-BSP2, respectively (Table 2 and Fig. 9b), which further confirmed the influence of the His-segments on the pH-sensitivity.^44^

### Evaluations of Dox-loaded NPs

The EE and LC of Dox-loaded NPs barely changed with the increasing of His segments. At the drug/carrier ratio of 1 : 5, the EE was approximately 80% at a theoretical LC of 10% for the His-SA-BSP1 and was 12% for the His-SA-BSP2. In a reported work, a silymarin-loaded BSP NPs showed the EE of 78% with LC of 7%.^19^ Furthermore, Guan *et al.* prepared a series of docetaxel-loaded BSP micelles.^20,21,45^ With different drug/carrier ratios, the EE were ranged from 70 to 90%, and LC were from 4 to 15%, which was in agreement with our results.

#### XRD evaluation

**Fig. 10:**
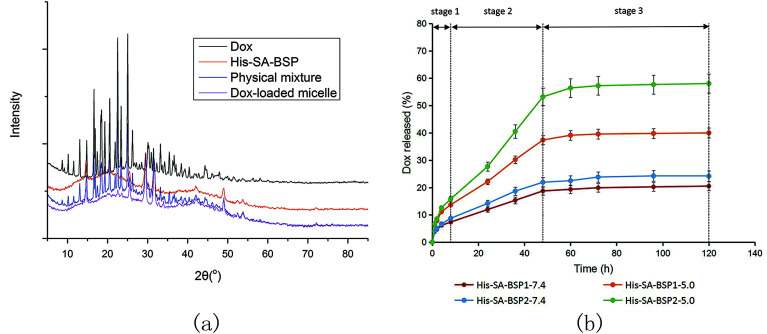
Characterizations of Dox-loaded NPs. (a) X-ray diffraction analysis; (b) Dox released profiles from His-SA-BSP micelles at different pH values (*n* = 3).

The XRD curves of His-SA-BSP showed broad peaks (Fig. 10a), indicating an amorphous material.^23^ The major peaks of the crystalline Dox (at 2*θ* range of 15° to 30°) were observed for the Drug powder and the physical mixture respectively. However, the characteristic peaks were disappeared in the drug-loaded NPs, suggesting that the drug had been successfully encapsulated into the NPs at the level of molecular dispersion.^46^

#### Dox-released profile

Fig. 10b shows the Dox release profiles from the NPs. The entire release process could be divided into three phase (Fig. 10b and Table 3): the fast release (0 to 8 hours), the zero-order release with a constant release rate (8 to 48 hours), and the equilibrium (48 to 120 hours).

**Table 3:** Parameters for the *in vitro* Dox-release profiles from His-SA-BSP NPs in different pH conditions (*n* = 3, mean ± S.D)

	pH 7.4				pH 5.0			
	Release rate (μg h^−1^)			Release percentage (%)	Release rate (μg h^−1^)			Release percentage (%)
	Stage 1	Stage 2	Stage 3		Stage 1	Stage 2	Stage 3	
His-SA-BSP1	1.584 ± 0.172	0.469 ± 0.051	0.042 ± 0.003	20.512 ± 1.641	2.894 ± 0.215	1.002 ± 0.064	0.0608 ± 0.004	39.98 ± 1.799
His-SA-BSP2	1.876 ± 0.155	0.567 ± 0.078	0.053 ± 0.005	24.159 ± 1.087	3.340 ± 0.192	1.579 ± 0.108	0.114 ± 0.011	58.04 ± 3.489

To verify the pH-responsive release profile as expected, the release behaviors of Dox from NPs under different pH environments were tested. The DOX cumulative releases from His-SA-BSP1 at pH values of 5.0 and 7.4 were 39.98% and 20.51% respectively, indicating that the acidic environments contributed to fast release rates. Additionally, the released percentage from His-SA-BSP2 was 58.04% (pH 5.0), further confirming the influences of His segment on the pH sensitivity. The interactions between the drug and carriers also contributed to the release efficiency. In the reported works,^19,21^ docetaxel and silymarin showed faster release profiles from BSP micelles than Dox, implying that Dox might have relatively high affinity to BSP.

In particular, the drug release mechanisms from pH-sensitive NPs can be classified into the following four types (1) the destruction of the amphipathic structures by the protonation or deprotonation of the polymers;^47^ (2) the separations of the polymer micelle amphiphilic blocks;^48^ (3) the expansions of the NPs related to the reduced hydrophobicity of the hydrophobic fragments;^49^ and (4) the breakage of the acid-labile bonds between the drugs and the NPs.^50^ As described in the previous section, the acidic environments boosted the protonation degree of imidazole, resulting in the expansion and destruction of the NP. Subsequently, the encapsulated Dox were released into the aqueous solutions.

### Cytotoxicity assay

**Fig. 11:**
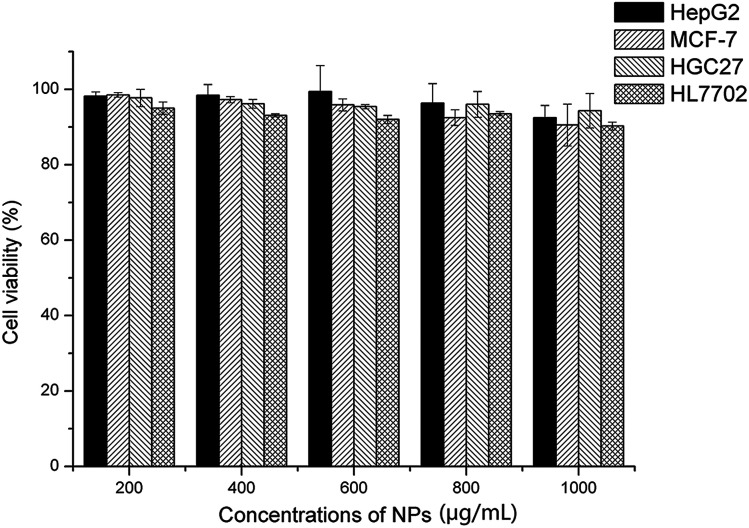
Cytotoxicity of blank NPs (His-SA-BSP2) towards different cell lines (*n* = 3).

Four different cell lines, including three tumor cells (HepG2, MCF-7, HGC-27) and a normal cell (HL-7702), were employed to evaluate the cytotoxicity of the empty NPs, as shown in Fig. 11. The cell viability remained over 90% after incubation for 48 hours, even though the NPs concentration reached 1000 μg mL^−1^. In general, the cytotoxicity of the NPs was usually along with the length of the alkyl chain and the surface positive charge. In this work, the grafted BSP presented a medium length of alkyl chain at the SA segments, and the His group provided the negative charge at the normal pH level. As a result, the formed NPs showed little cytotoxicity. Furthermore, this finding was supported by Wang^18^ and Guan^21^ in their reported works.

### Tumor cell inhibition

**Fig. 12:**
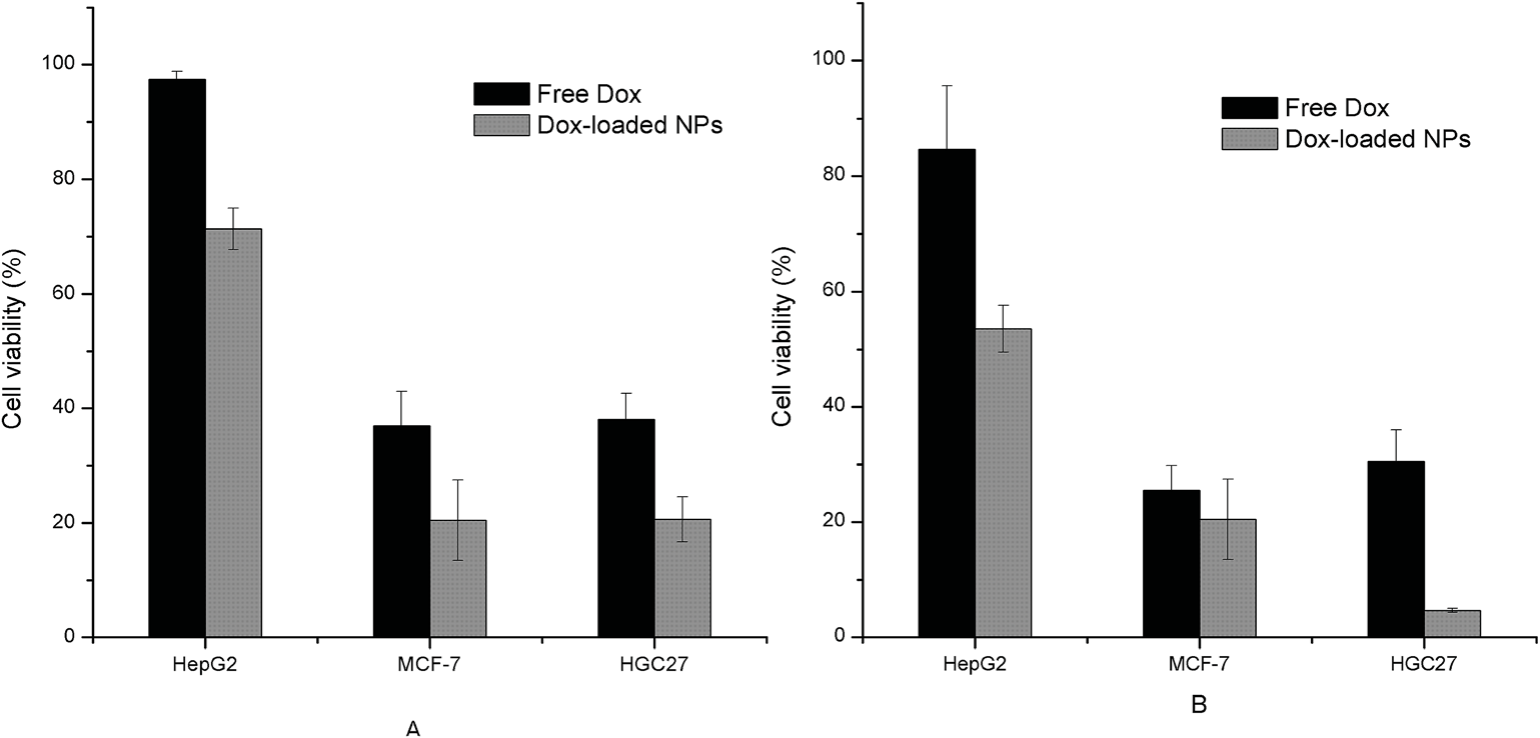
Tumor cell inhibition of free Dox and Dox-loaded NPs (His-SA-BSP2) with different concentration (*n* = 3). (A) The concentration of NPs was 41.67 μg mL^−1^, containing 5 μg mL^−1^ Dox; (B) the concentration of NPs was 83.33 μg mL^−1^, containing 10 μg mL^−1^ Dox. The concentration of the free Dox was set at 5 μg mL^−1^ (a) and 10 μg mL^−1^ (b), respectively.

Both the free Dox and the Dox-loaded NPs showed inhibition effects on the three tumor cells with a dose-dependent manner, as shown in Fig. 12. The IC_50_ values of free Dox and Dox-loaded NPs towards each cell line were also calculated and listed in Table 4. It was observed from the results that the cell inhibition of NPs group had statistically difference compared with that of free Dox. The pH-sensitive NPs with small size were liable to directly penetrate into tumor cells through endocytosis, which suggested an enhancement against tumor cells. Furthermore, the differences in cell viability and IC_50_ observed among the three model cells may attributed to the differences in their cell physiologies and sensitivities.^20^

**Table 4:** IC_50_ of free Dox and Dox loaded NPs towards different tumor cell lines (*n* = 3, mean ± S.D)^a^

Group	IC_50_ (μg mL^−1^)		
	HepG2	MCF-7	HGC27
Free Dox	115.31 ± 8.89^a^	4.02 ± 0.11^b^	3.23 ± 0.07^c^
Dox-loaded NPs	243.75 ± 12.57^d^	32.51 ± 2.31^e^	20.51 ± 1.67^f^

a The upper character suggested the statistic differences from other groups (*p* < 0.05). The Dox loading content was 12%.

### Cell uptake

#### Fluorescence microscopy

**Fig. 13:**
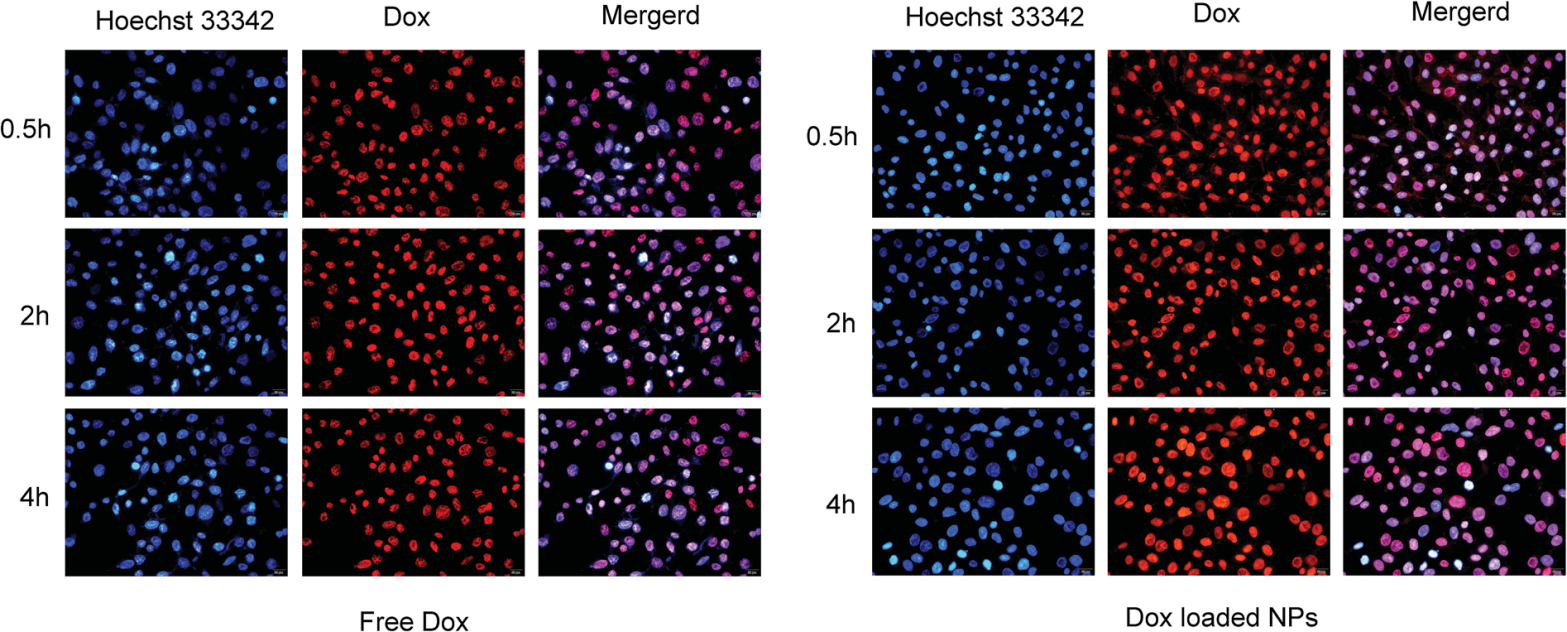
Fluorescence microscopy images of MCF-7 cells incubated with free Dox and Dox-loaded NPs (His-SA-BSP2). Blue and red colors indicate Hoechst 33 342 and Dox respectively. The Dox concentration was set as 10 μg mL^−1^ for both free Dox and Dox loaded NPs.

The cell uptake of free Dox and Dox-loaded NPs was investigated by fluorescence microscopy towards MCF-7 cells, as shown in Fig. 13. Since DOX possesses spontaneous red fluorescence, the DOX-NPs did not require labelling by any other luminescent dyes.^51^ It was observed that the fluorescence intensity of Dox increased over the incubation time, and reached the strongest at 4 hours, demonstrating a time-dependent manner. In contrast, NPs displayed stronger fluorescence of Dox in MCF-7 cells for the same interval, indicating that Dox in His-SA-BSP2 NPs had an improved cell uptake, which might be attributed to the pH sensitivity of the carriers. The NPs could reverse its surface charge to facilitate cellular uptake in response to the extracellular pH environment. On entry into the tumor cells, the NPs could escape quickly from lysosomes. The acidic intracellular microenvironment triggered the expansion and destruction of the NP, resulting in the rapidly release of Dox into the cytoplasm.^25^

#### Flow cytometry

**Fig. 14:**
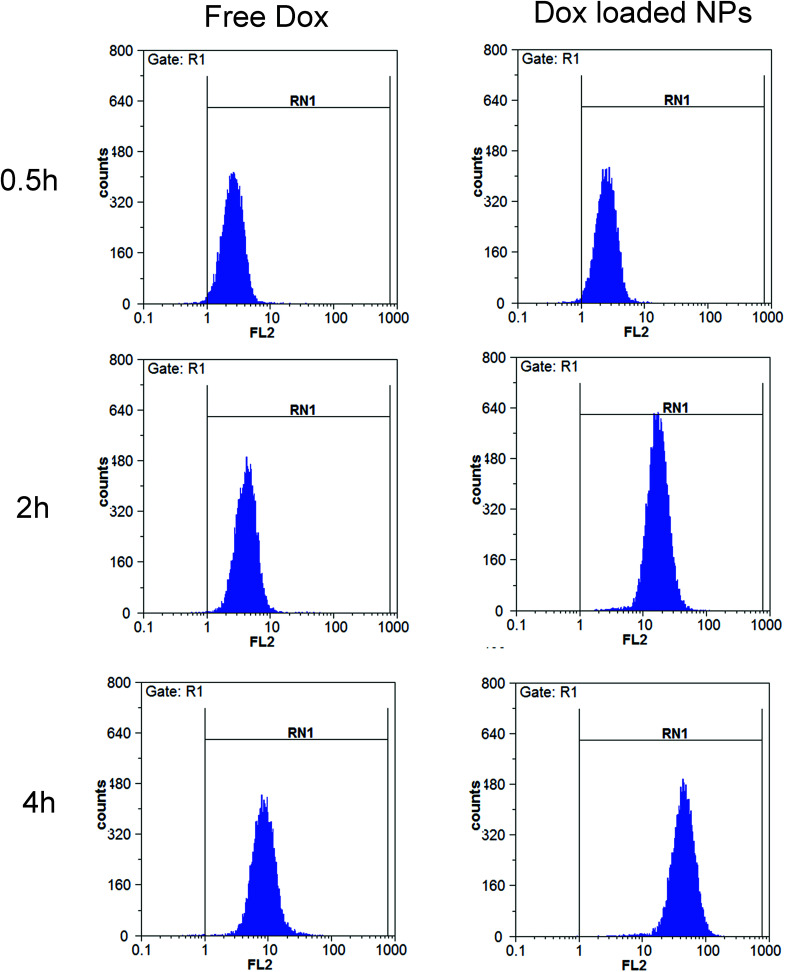
Flow cytometry measurement of the cellular uptake of free Dox (10 μg mL^−1^) and Dox-loaded NPs (containing 10 μg mL^−1^ Dox) in MCF-7 cells (*n* = 3).

**Fig. 15:**
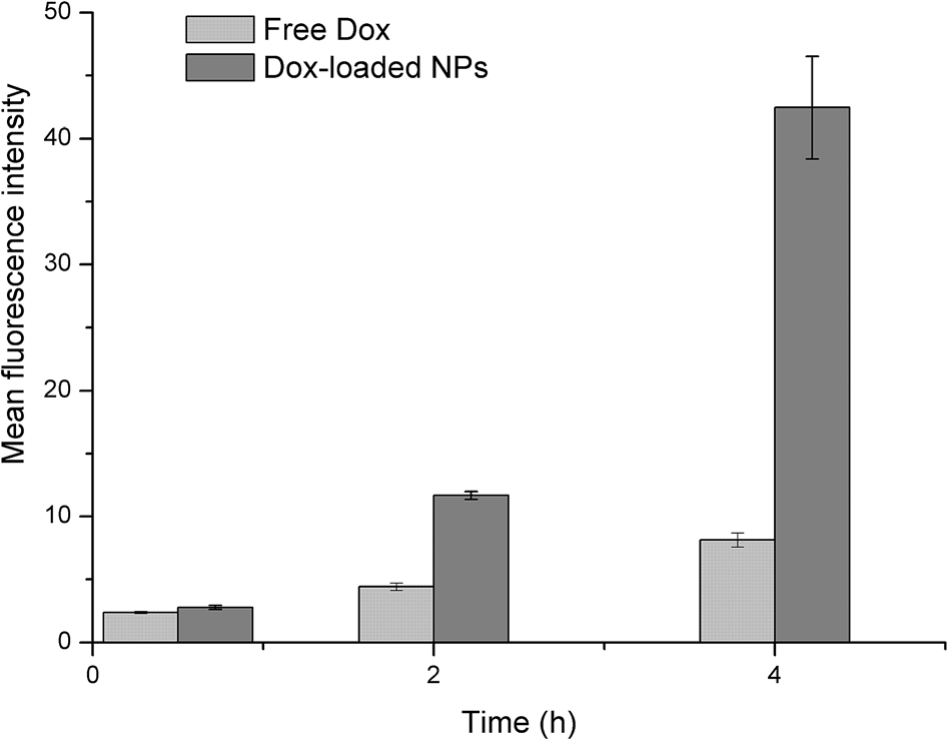
Mean fluorescence intensity of Dox in MCF-7 cells, as calculated by flow cytometry (*n* = 3).

The cellular uptakes were also analyzed quantitatively using flow cytometry, as shown in Fig. 14. The increased fluorescence intensity in MCF-7 was also time dependent and the Dox-loaded NPs had a larger value at each time point. The cell uptake of Dox in BSP NPs was 2.63- and 5.22-fold higher than that of free Dox at 2 and 4 hours respectively (Fig. 15), implying that the NPs could release the drug rapidly after internalizing into the MCF-7 cells, which was in agreement with the result of fluorescence microscopy assay.

## Conclusion

In the current work, novel pH-sensitive and amphiphilic His-SA-PS were successfully synthesized. The His-SA-BSP was found to have the ability to self-assemble into NPs in neutral solutions with sizes of approximately 200 nm. The CMC was found to range from 37 to 71 μg mL^−1^. It was observed that the acidic condition could trigger the expansion of the particle sizes (400 nm) and reverse the surface charge, which were related to the His segment. The acidic condition also boosted the release profiles of Dox from the NPs. The CCK-8 assay demonstrated a good biocompatibility of the carriers towards different cell lines and a specific inhibition effect of Dox-loaded NPs against tumor cells. Moreover, the NPs showed an enhancement on cellular uptake of Dox towards MCF-7 by fluorescence microscopy and flow cytometry. All of the aforementioned attributes implied that the His-SA-BSP displayed promising future potential applications in targeted and smart drug delivery systems.

## Conflicts of interest

There are no conflicts of interest to declare.

## Supplementary Material

## References

[cit1] Yu Y., Shen M., Song Q., Xie J. (2018). Carbohydr. Polym..

[cit2] Wang Y., Li P., Chen F., Jia L., Xu Q., Gai X., Yu Y., Di Y., Zhu Z., Liang Y., Liu M., Pan W., Yang X. (2017). Sci. Rep..

[cit3] Alvarez-Lorenzo C., Blanco-Fernandez B., Puga A. M., Concheiro A. (2013). Adv. Drug Delivery Rev..

[cit4] Kanamala M., Wilson W. R., Yang M., Palmer B. D., Wu Z. (2016). Biomaterials.

[cit5] Swierczewska M., Han H. S., Kim K., Park J. H., Lee S. (2016). Adv. Drug Delivery Rev..

[cit6] Mura S., Nicolas J., Couvreur P. (2013). Nat. Mater..

[cit7] Ganguly K., Chaturvedi K., More U. A., Nadagouda M. N., Aminabhavi T. M. (2014). J. Controlled Release.

[cit8] Wang Y., Liu D., Chen S., Wang Y., Jiang H., Yin H. (2014). Fitoterapia.

[cit9] Ye Y., Chou G.-X., Mu D.-D., Wang H., Chu J.-H., Leung A. K.-M., Fong W.-f., Yu Z.-L. (2010). J. Ethnopharmacol..

[cit10] Dong L., Xia S., Luo Y., Diao H., Zhang J., Chen J., Zhang J. (2009). J. Controlled Release.

[cit11] Wang C., Sun J., Luo Y., Xue W., Diao H., Dong L., Chen J., Zhang J. (2006). Biotechnol. Lett..

[cit12] Wang Y., Liu J., Li Q., Wang Y., Wang C. (2015). Biotechnol. Lett..

[cit13] Liu K., Feng Z., Shan L., Yang T., Qin M., Tang J., Zhang W. (2017). Drying Technol..

[cit14] Luo Y., Diao H., Xia S., Dong L., Chen J., Zhang J. (2010). J. Biomed. Mater. Res., Part A.

[cit15] Liu J. Y., Wang H. C., Yin Y., Li N., Cai P. L., Yang S. L. (2012). Carbohydr. Polym..

[cit16] Zhan X., Jia L., Niu Y., Qi H., Chen X., Zhang Q., Zhang J., Wang Y., Dong L., Wang C. (2014). Biomaterials.

[cit17] Zhang M., Sun L., Zhao W., Peng X., Liu F., Wang Y., Bi Y., Zhang H., Zhou Y. (2014). Molecules.

[cit18] Wang W., He S., Hong T., Zhang Y., Sui H., Zhang X., Ma Y. (2017). Artif. Cells, Nanomed., Biotechnol..

[cit19] Ma Y., He S., Ma X., Hong T., Li Z., Park K., Wang W. (2016). Molecules.

[cit20] Zhao L., Sun D., Lu H., Han B., Zhang G., Guan Q. (2018). J. Pharm. Pharmacol..

[cit21] Guan Q., Sun D., Zhang G., Sun C., Wang M., Ji D., Yang W. (2016). Molecules.

[cit22] Huang Z., Gan J., Long Z., Guo G., Shi X., Wang C., Zang Y., Ding Z., Chen J., Zhang J., Dong L. (2016). Biomaterials.

[cit23] Cui X., Zhang X., Yang Y., Wang C., Zhang C., Peng G. (2017). Pharm. Dev. Technol..

[cit24] Debele T. A., Mekuria S. L., Tsai H. C. (2017). Int. J. Biol. Macromol..

[cit25] Qiu L., Li Z., Qiao M., Long M., Wang M., Zhang X., Tian C., Chen D. (2014). Acta Biomater..

[cit26] Chen Z., Xin M., Li C. (2013). Polym. Mater. Sci. Eng..

[cit27] Li Y., Yuan Y., Lei L., Li F., Zhang Y., Chen J., Zhao G., Wu S., Yin R., Ming J. (2017). Carbohydr. Polym..

[cit28] Wu Y., Ye M., Du Z., Jing L., Surahio M., Yang L. (2014). Carbohydr. Polym..

[cit29] Dos Santos Z. M., Caroni A. L., Pereira M. R., da Silva D. R., Fonseca J. L. (2009). Carbohydr. Res..

[cit30] Calce E., Mercurio F. A., Leone M., Saviano M., De Luca S. (2016). Carbohydr. Polym..

[cit31] Wu X., Chen X., Hu P., Hou M., Dong Y., Wei Y. (2018). Carbohydr. Polym..

[cit32] Lin W., He Y., Zhang J., Wang L., Wang Z., Ji F., Chen S. (2014). Colloids Surf., B.

[cit33] Campos D., Chirinos R., Barreto O., Noratto G., Pedreschi R. (2013). Ind. Crops Prod..

[cit34] Silva D. A., de Paula R. C. M., Feitosa J. P. A., de Brito A. C. F., Maciel J. S., Paula H. C. B. (2004). Carbohydr. Polym..

[cit35] Jafarzadeh-Holagh S., Hashemi-Najafabadi S., Shaki H., Vasheghani-Farahani E. (2018). J. Colloid Interface Sci..

[cit36] Lee E. S., Gao Z., Kim D., Park K., Kwon I. C., Bae Y. H. (2008). J. Controlled Release.

[cit37] Kong L., Yu L., Feng T., Yin X., Liu T., Dong L. (2015). Carbohydr. Polym..

[cit38] Yang X., Shi X., D'Arcy R., Tirelli N., Zhai G. (2018). J. Controlled Release.

[cit39] Kumar Varma C. A., Jayaram Kumar K. (2017). Carbohydr. Polym..

[cit40] Sun Y., Li Y., Nan S., Zhang L., Huang H., Wang J. (2015). J. Colloid Interface Sci..

[cit41] Yoo W., Yoo D., Hong E., Jung E., Go Y., Singh S. V. B., Khang G., Lee D. (2018). J. Controlled Release.

[cit42] Wu H., Zhu L., Torchilin V. P. (2013). Biomaterials.

[cit43] Cho H. J., Yoon H. Y., Koo H., Ko S. H., Shim J. S., Lee J. H., Kim K., Kwon I. C., Kim D. D. (2011). Biomaterials.

[cit44] Tang B., Zaro J. L., Shen Y., Chen Q., Yu Y., Sun P., Wang Y., Shen W. C., Tu J., Sun C. (2018). J. Controlled Release.

[cit45] Guan Q., Zhang G., Sun D., Wang Y., Liu K., Wang M., Sun C., Zhang Z., Li B., Lv J. (2017). PLoS One.

[cit46] Mukhopadhyay P., Maity S., Mandal S., Chakraborti A. S., Prajapati A. K., Kundu P. P. (2018). Carbohydr. Polym..

[cit47] Gao G. H., Li Y., Lee D. S. (2013). J. Controlled Release.

[cit48] Zhang Y., Li P., Pan H., Liu L., Ji M., Sheng N., Wang C., Cai L., Ma Y. (2016). Biomaterials.

[cit49] Chen W., Meng F., Cheng R., Zhong Z. (2010). J. Controlled Release.

[cit50] Gu Y., Zhong Y., Meng F., Cheng R., Deng C., Zhong Z. (2013). Biomacromolecules.

[cit51] Yang S., Tang Z., Zhang D., Deng M., Chen X. (2017). Biomater. Sci..

